# Peptidoglycan Muropeptides: Release, Perception, and Functions as Signaling Molecules

**DOI:** 10.3389/fmicb.2019.00500

**Published:** 2019-03-28

**Authors:** Oihane Irazoki, Sara B. Hernandez, Felipe Cava

**Affiliations:** Laboratory for Molecular Infection Medicine Sweden, Department of Molecular Biology, Umeå Centre for Microbial Research, Umeå University, Umeå, Sweden

**Keywords:** peptidoglycan, PG cleaving enzymes, PG recycling, PG receptors, signaling functions, bacterial interactions

## Abstract

Peptidoglycan (PG) is an essential molecule for the survival of bacteria, and thus, its biosynthesis and remodeling have always been in the spotlight when it comes to the development of antibiotics. The peptidoglycan polymer provides a protective function in bacteria, but at the same time is continuously subjected to editing activities that in some cases lead to the release of peptidoglycan fragments (i.e., muropeptides) to the environment. Several soluble muropeptides have been reported to work as signaling molecules. In this review, we summarize the mechanisms involved in muropeptide release (PG breakdown and PG recycling) and describe the known PG-receptor proteins responsible for PG sensing. Furthermore, we overview the role of muropeptides as signaling molecules, focusing on the microbial responses and their functions in the host beyond their immunostimulatory activity.

## Introduction

Most bacteria surround themselves with a protective cell wall to repel environmental challenges. These tough cell walls are primarily composed of a peptidoglycan (PG) exoskeleton, also called the murein sacculus (Vollmer et al., 2008a; de Pedro and Cava, 2015). PG is a highly dynamic macromolecule subjected to constant remodeling in response to changing environmental conditions (Horcajo et al., 2012). It counteracts osmotic pressure, maintains cell shape and integrity, and serves as a protective barrier against physical, chemical, and biological threats (Holtje, 1998; Vollmer et al., 2008a). PG is found on the outside of the cytoplasmic membrane of almost all bacteria (Nanninga, 1998; Mengin-Lecreulx and Lemaitre, 2005) and presents a conserved overall composition and biogenesis, although the complexity and thickness of the structure vary (Cava and de Pedro, 2014). Peptidoglycan also serves as a scaffold for anchoring other cell envelope components such as proteins (Dramsi et al., 2008) and teichoic acids (Neuhaus and Baddiley, 2003).

**Figure 1:**
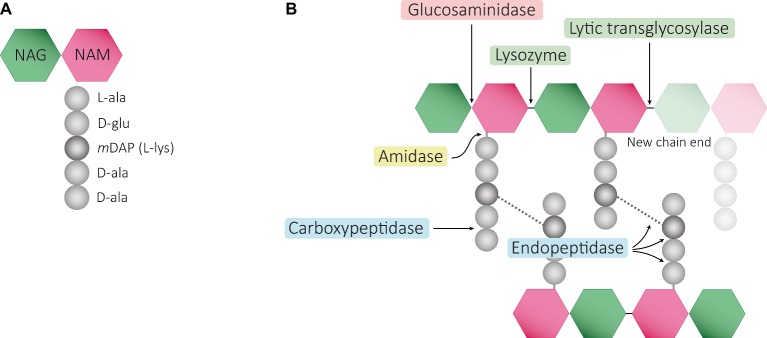
Schematic representation of muropeptides and peptidoglycan. **(A)** The archetypical structure of muropeptides consist of NAG-NAM disaccharides attached to a peptide chain containing 2- to 5 amino acid residues, typically: L-alanine, D-glutamic acid, *m*DAP/L-Lys, D-alanine, and D-alanine. **(B)** Diverse cleavage points of PG cleaving enzymes: glucosaminidases (pink), amidases (yellow), peptidases (blue), and muramidases (green) are shown.

Structurally speaking, the PG sacculus is made up of linear glycan strands cross-linked to each other by short peptide chains forming a continuous layer. The glycan backbone generally consists of repeating disaccharides of *N*-acetylglucosamine (NAG) and *N*-acetylmuramic acid (NAM) covalently attached to a peptide chain containing 2–5 amino acid residues. The archetypical peptide stem structure is L-alanine, D-glutamic acid, a dibasic amino acid [typically meso-diaminopimelic acid (*m*DAP) or L-lysine], D-alanine, and D-alanine (Figure 1A). Some of the peptide chains from adjacent glycan strands are cross-linked, resulting in a thick three-dimensional multi-layered meshwork. This arrangement is widely conserved across most bacterial species; however, the chemistry of the residues of the peptide stem, the glycan chains, and the type of crosslinking can vary (Vollmer et al., 2008a). These variations alter the properties of the cell wall and allow for great diversity in fine structure and architecture (Schleifer and Kandler, 1972; Vollmer and Bertsche, 2008; Cava and de Pedro, 2014; Turner et al., 2014). For more detailed information about PG structure, synthesis, and regulation, we refer to extended reviews (Vollmer et al., 2008a; Typas et al., 2011; Egan et al., 2017).

During growth and maturation, PG is degraded by dedicated enzymes, which shed PG fragments (or muropeptides) in a process termed PG turnover. In *E. coli*, in a single generation of growth, as much as 50% of the PG is excised from the cell wall as anhydromuropeptides, suggesting a robust turnover of the cell wall (Doyle et al., 1988). Around the 95% of these are efficiently recovered and reused through the PG-recycling pathway (Goodell and Schwarz, 1985; Park and Uehara, 2008).

In recent years, PG has been of much interest not only because it is one of the major antibiotic targets (Kohanski et al., 2010) but also due to its importance in host physiology and metabolism since it presents immunostimulatory activities (Girardin et al., 2003a,b). Some PG-derived fragments are recycled for cell wall biosynthesis but they are also used in bacterial communication and are detected by eukaryotes to initiate an immune response (Girardin et al., 2003a,b; Boudreau et al., 2012; Woodhams et al., 2013; Dworkin, 2014). Recent data suggest that muropeptides have many diverse roles, including involvement in symbiotic associations, microbial interactions, and pathogenesis in animals and plants. In this review, we focus on the signaling functions of PG fragments, describing the mechanisms involved in the release of these molecules and the means by which they are sensed by bacterial and host cells.

## Muropeptides Release

It is well documented that the PG sacculus is remodeled during bacterial growth and that this process causes the release of muropeptides. The discharge of PG fragments can occur as a consequence of the disruption of PG during growth or by the complete lysis of cells.

### PG Cleaving Enzymes

Cleavage of PG is required for fundamental physiological processes in bacteria such as enlargement of the PG sacculus during bacterial growth and cell separation during cell division (Holtje, 1998; Layec et al., 2008; Uehara et al., 2010; Typas et al., 2011; Uehara and Bernhardt, 2011; Waldemar, 2012); incorporation and assembly of protein complexes into the cell wall (e.g., secretion, conjugation, and flagellum systems) (Dijkstra and Keck, 1996; Koraimann, 2003; Scheurwater et al., 2008; Scheurwater and Burrows, 2011; Stohl et al., 2013); or sporulation and resuscitation of dormant states (Keep et al., 2006; Wyckoff et al., 2012; Popham and Bernhards, 2015). Enzymes cleaving the bonds that exist within PG are generally known as PG hydrolases (PGHs), and although some (i.e., lytic transglycosylases) do not present chemical hydrolytic activity, from now on we will refer to all them as PGHs. Despite the large number and diversity of proteins cleaving the PG, they can be grouped accordingly to the type of the bond cleaved such as glycosidases (cleaving glycosidic bonds of the glycan strands), amidases (hydrolyzing the amide bond between the first amino acid of the stem peptide and the NAM), and peptidases (cleaving bonds between amino acids present in the stem peptides) (Figure 1B). They often act on a particular type of PG, cleaving intact high-molecular-weight murein sacculi and its soluble fragments (Vollmer et al., 2008b).

PG glycan chains contain two glycosidic bonds sensitive to the activity of glycosidases: the bond between a NAG and the adjacent NAM is hydrolyzed by *N-*Acetyl-β-glucosaminidases (*N*-acetylglucosaminidases), while muramidases (or muralytic enzymes) cleave the bond between sequential NAM and NAG residues (Figure 1B). Muramidases are divided into two subgroups depending on their catalytic mechanism: lysozymes are hydrolytic enzymes that add water across the glycosidic bond during the cleavage generating a reducing NAM product; while lytic transglycosylases (LTs) catalyze an intramolecular rearrangement involving the C-6 hydroxyl group of the NAM resulting in the formation of unique 1,6-anhydro-*N-*acetylmuramic acid products, the so-called anhydromuropeptides (Holtje et al., 1975; Thunnissen et al., 1995; Callewaert and Michiels, 2010). PG peptidases can be classified into two groups: carboxypeptidases (removing the C-terminal amino acid of peptide stems) and endopeptidases (cleaving within the peptide cross-links), and both can be referred to as DD-, LD-, or DL-peptidases based on the isomeric form of the two amino acids that are split (Vollmer et al., 2008b).

PGHs are ubiquitous among all eubacteria (Shockman et al., 1996; Firczuk and Bochtler, 2007; Layec et al., 2008; Sharma et al., 2016). Many species present a large number of PG cleaving enzymes, and while for some of them, functional redundancy has been observed under laboratory conditions (van Heijenoort, 2011; Rolain et al., 2012; Singh et al., 2012), and specific functions have been demonstrated for others (Schaub et al., 2016; Santin and Cascales, 2017). During growth, PGHs are capable of fulfilling the PG-remodeling demands acting on the murein sacculus without disrupting the structural integrity of the cell wall, but a regulatory failure of their activity could easily lead to uncontrolled PG degradation and consequent cell lysis (autolysis) (van Heijenoort, 2011). Therefore, bacterial PGHs (so-called autolysins) must be regulated in order to prevent accidental lysis (Rice and Bayles, 2008). Regulation of bacterial PGH activity has been characterized at different levels including gene expression, subcellular localization, the formation of multi-enzyme catalytic complexes, or modification of the PG substrate (Holtje and Tuomanen, 1991; Vollmer et al., 2008b; Chapot-Chartier, 2010; Morlot et al., 2010).

The cleavage of covalent bonds in the murein sacculus during cell wall metabolism leads to the release of PG-derived material. Depending on the cleaving enzyme, different fragments can be released from the PG sacculus: both lysozymes and lytic transglycosylases release disaccharide-peptides, but while the hydrolytic reaction of lysozymes generates a terminal reducing NAM (Callewaert and Michiels, 2010), lytic transglycosylases produce anhydromuropeptides which present a 1,6-anhydro ring at the NAM (anhNAM) (Holtje et al., 1975); monosaccharide-peptides (named here muramyl peptides) can also be produced by the activity of *N-*acetylglucosaminidases (Votsch and Templin, 2000). Even though peptidase or amidase activities do not release muropeptides or muramyl-peptides themselves, their role remodeling the high-molecular-weight murein sacculus or its soluble fragments (e.g., amidase activity releases NOD1-stimulatory free peptides) certainly shape the number and the chemical composition of the released molecules (Lenz et al., 2016).

**Figure 2:**
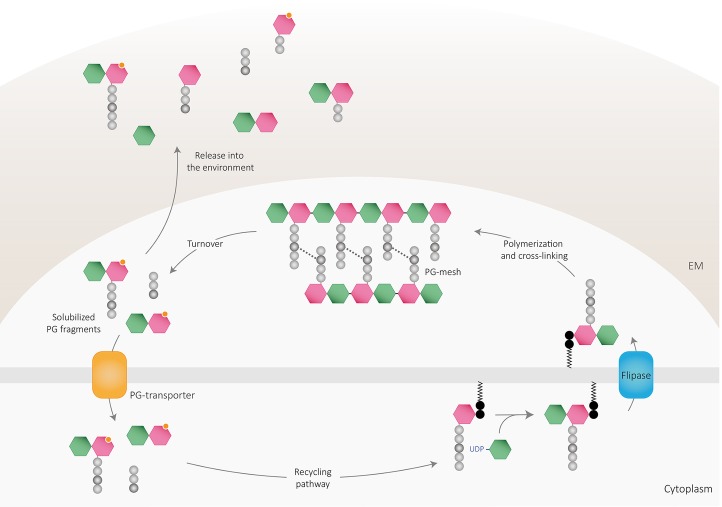
Peptidoglycan recycling and muropeptide release. PG cleaving enzymes digest the sacculi delivering PG fragments to the periplasm, which can be either released to the environment or transported into the cytoplasm through PG transporters. Once in the cytosol, PG fragments might enter the recycling pathway to finally be reincorporated into the newly polymerized PG mesh or used as an own-energy source by the cell. Part of PG-turnover products is released to the environment, where are detected by other cells and can act as signaling molecules. EM: extracellular matrix.

PG fragments are solubilized from the murein sacculus in active bacteria by a process termed cell wall turnover (Figure 2) that leads to the excision of muropeptides, shedding, and cell wall catabolism (Chaloupka, 1962; Doyle et al., 1988). As mentioned, up to half of the pre-existing PG is turned over and discharged from the wall every generation in both Gram-positive and Gram-negative bacteria (Mauck et al., 1971; Wong et al., 1974; Dworkin, 2014). The released material can be reimported into the bacterial cytoplasm and reused for PG synthesis or as nutrient or energy sources through an efficient PG recycling pathway (Chaloupka and Strnadova, 1972; Goodell and Schwarz, 1985; Park and Uehara, 2008; Borisova et al., 2016) or liberated to the environment. Accordingly, the bacterial PG recycling pathway modulates to some extent the bioavailability of soluble fragments (Johnson et al., 2013).

### PG Recycling

Historically, it has been assumed that PG recycling was limited to Gram-negative bacteria since, in comparison, larger amounts of PG turnover products were isolated from the growth medium of several Gram-positives (Mauck et al., 1971). Nevertheless, orthologs of some recycling enzymes are present in most Gram-positive bacteria (Park and Uehara, 2008; Litzinger et al., 2010; Reith and Mayer, 2011). In fact, recent studies have shown that PG recycling also occurs in different Gram-positives (Borisova et al., 2016; Kluj et al., 2018), although reuse of PG sugars and peptide turnover products for murein synthesis in these organisms is currently unclear. PG recycling has been more extensively studied in Gram-negative bacteria (Johnson et al., 2013; Dhar et al., 2018) where recycling begins with degradation of the PG by the activity of PGHs. LTs are the main enzymes involved in high-molecular-weight sacculus degradation and therefore play a key role in PG recycling (Dominguez-Gil et al., 2016).

Most bacteria encode multiple LTs (e.g., 8 have been described in *E. coli* and 11 in *P. aeruginosa*), which can be divided into soluble periplasmic LTs (named Slts) or membrane-attached LTs (named Mlts), and can perform the cleavage at the end of the glycan strands (exolytic) and/or in the middle of the PG chains (endolytic) (Dik et al., 2017). Although redundancy in generating soluble anhydromuropeptides has been observed by single and multiple deletion analysis (Korsak et al., 2005; Lamers et al., 2015), a unique contribution from some hydrolytic enzymes has also been proven (Kraft et al., 1999). Particularly, Slt70 of *E. coli* is considered to be the major LT involved in PG-turnover, as it has been shown to be the main enzyme following β-lactam treatment (Cho et al., 2014).

Depending on the efficiency and regulation of the PG-recycling pathway of the bacterium, anhydromuropeptides can either be transported to the cytoplasm (where they are subsequently processed by the activity of several enzymes) or released to the environment by a currently unknown mechanism (Figure 2). In *E. coli*, the gate of entry for the internalization of soluble anhydromuropeptide monomers (NAG-anhNAM-peptides) into the cytoplasm is the AmpG permease, an inner transmembrane protein that specifically takes up anhydromuropeptides or free anhydrodisaccharides (Cheng and Park, 2002). Deletion of the gene encoding AmpG prevents the uptake of anhydromuropeptides leading to their accumulation in the medium (Jacobs et al., 1994; Wiedemann et al., 1998; Garcia and Dillard, 2008; Nyholm, 2009) revealing the importance of recycling as a limiting factor for PG-fragment release. Once in the cytosol, anhydromuropeptides are further hydrolyzed by a mechanism involving a set of dedicated enzymes (extensively reviewed in Johnson et al., 2013; Dhar et al., 2018). The specific activities of NagZ (*N-*acetylglucosaminidase) and AmpD (*N-*L-alanine amidase) on molecules presenting anhNAM structure yield NAG, anhNAM, and free peptides in the cytoplasm (Holtje et al., 1994; Cheng et al., 2000; Votsch and Templin, 2000; Lee et al., 2009). Resulting tetrapeptides are hydrolyzed by the action of the L,D-carboxypeptidase LdcA (Templin et al., 1999) into tripeptides, which can be degraded into individual amino acids for utilization as nutrient or energy sources (Schroeder et al., 1994; Schmidt et al., 2001; Uehara and Park, 2003) or attached directly to UDP-NAM by the murein peptide ligase Mpl (Mengin-Lecreulx et al., 1996; Das et al., 2011). Ligated UDP-NAM-tripeptides and processed sugar products can then be recycled by entering the pathway for *de novo* PG synthesis (White and Pasternak, 1967; Uehara et al., 2005, 2006).

The internalization of anhydromuropeptides and subsequent breakdown in order to supply a demanding nutrient or energy sources seems unlikely under favorable growth conditions (Uehara and Park, 2008), and even if enzymes involved in PG degradation are expressed during growth (Park and Uehara, 2008; Maqbool et al., 2012), it has been estimated that 97% of the recovered material is reutilized for new PG synthesis (Goodell, 1985). Furthermore, even if the switching control between PG recycling and catabolism is not clear yet, some genes encoding for enzymes involved in degradation of the peptide have been shown to be de-repressed under nutrient starvation (Shimada et al., 2013) pointing out a tight control between these two processes.

### Distribution and Function(s) of PG Recycling

While PG turnover is widespread in bacteria, it remains unclear how prevalent PG recycling is. Since this pathway relies upon the transport of anhydromuropeptides into the cytosol, the existence of AmpG-like permeases may be required for PG recycling. Though PG recycling has only been experimentally proven in certain species, AmpG is present in diverse Gram-negatives (Uehara and Park, 2008) but apparently absent in Gram-positives (Reith and Mayer, 2011). This observation may fit with a limited number of known LTs in Gram-positives (Dik et al., 2017) and their apparently restricted function to PG enlargement (Tsui et al., 2016), spore formation/germination (Heffron et al., 2009), or induction of autolysis (Wydau-Dematteis et al., 2018). On the other hand, abundant lysozyme-like enzymes, *N*-acetylglucosaminidases and amidases, have been described to act at the cell wall compartment in Gram-positive bacteria (Lopez et al., 1997; Smith et al., 2000; Vollmer and Bertsche, 2008). These activities liberate PG fragments presenting terminal-reducing NAM and free peptides, which can be taken up by other specific transporters, offsetting the lack of an AmpG permease (Reith and Mayer, 2011).

The presence of orthologs of other genes involved in the pathway also points toward the existence of a dedicated route for recycling PG-degradation products. In this regard, genes involved in the processing and reutilization of PG sugars are widespread among both Gram-negative and Gram-positive bacteria (Jaeger and Mayer, 2008; Borisova et al., 2014), suggesting an extensive role and consequently important function(s) of PG recycling. Though the primary function of PG recycling is not clear (it is not essential under experimental conditions) (Jacobs et al., 1994; Cheng et al., 2000), it has been reported to be involved in a range of diverse processes. It is still widely thought that reutilization of PG fragments as carbon and energy sources is potentially critical to promote growth under nutrient-limiting conditions, but for *E. coli* there is no clear evidence supporting this hypothesis. Nevertheless, reutilization of PG recycled products has been observed to be essential in particular cases. The use of recycled NAM for cell wall synthesis apparently increases survival of *Bacillus subtilis* and *Staphylococcus aureus* under starvation conditions during stationary phase (Borisova et al., 2016), which is consistent with previous findings showing that in Gram-positive bacteria MurQ and NagZ expression is higher in stationary phase (Litzinger et al., 2010; Botella et al., 2011). In the Gram-negative oral anaerobe *Tannerella forsythia*, which is unable to synthesize its own PG sugars, scavenging environmental muropeptides (released by cohabiting bacteria) through an AmpG-like transporter is vital for PG-synthesis (Ruscitto et al., 2017). Additionally, in two Cyanobacteria species, PG recycling has been suggested to be an energy-saving strategy to promote growth under light-limiting conditions (Jiang et al., 2010).

Aside from the importance of the reutilization of PG products, other functions proposed for PG recycling are more related to the production and accumulation of solubilized cell wall fragments when the pathway is not working efficiently. In this regard, a variety of messenger functions have been attributed to PG fragments, which are compiled below.

### Alternative Ways to Produce Soluble PG Fragments

The essentiality and uniqueness of the bacterial PG make this structure an excellent antibacterial target (Kohanski et al., 2010; Muller et al., 2017). It is therefore not surprising that lysozymes, which exhibit a highly specific cleavage activity on PG, are widespread (Callewaert and Michiels, 2010). Although the antimicrobial action of lysozymes is also intimately related to their structure (Ibrahim et al., 2001), their catalytic activity disrupts PG by hydrolyzing the β-1,4 glycosidic bonds linking adjacent PG monomers, resulting in cell lysis and successive release of muropeptides. Production of lysozymes constitutes a natural defence mechanism against bacterial pathogens (Bertsche et al., 2015), and consequently, pathogenic bacteria have developed different mechanisms to evade lysozyme action such as modification of the PG (Yadav et al., 2018), alteration of the charge, and strength of the envelope or the production of lysozyme inhibitors (Ragland and Criss, 2017).

As a defence mechanism, plants and animals have exploited PG structure and developed mechanisms to monitor the presence of bacteria through PG recognition proteins (PGRPs) (Royet et al., 2011; Gust, 2015), some of which also present PG-cleavage activity separate from their ability to sense bacterial PG (Royet and Dziarski, 2007; Royet et al., 2011). These PGRPs present *N-*acetylmuramoyl-L-alanine amidase activity that hydrolyzes the amide bond between NAG and L-alanine in peptidoglycan and removes the stem peptides from the glycan chain, contributing to the release of PG fragments to the environment.

Furthermore, many Gram-negative bacteria can interact with other microbes and the host by releasing outer membrane vesicles (OMVs) to the environment (Kulp and Kuehn, 2010). Formation of OMVs has also been suggested as another mechanism of delivering peptidoglycan in several Gram-negative human pathogens (Kaparakis et al., 2010; Bielig et al., 2011).

## Detection of Released Muropeptides

Specific roles for a variety of soluble PG fragments as messenger molecules have been known for decades (Adam and Lederer, 1984) and have come into focus more recently. Microbe-associated molecular patterns (MAMPs) are defined as molecular signatures highly conserved in bacteria but absent from the host cells (Boller and Felix, 2009). MAMPs are detected by specific receptors termed pattern recognition receptors (PRRs) that are able to bind PG among other molecules (including lipopolysaccharides, lipoteichoic acids, lipoproteins, microbial DNA and RNA, flagellin, fungal cell wall glucans, or chitin) (Strober et al., 2006; Cinel and Opal, 2009; Mogensen, 2009; Diacovich and Gorvel, 2010). In the host, MAMP recognition leads to the activation of PRR-induced signal pathways that trigger the expression of a broad range of molecules, including adaptor molecules, cytokines, chemokines, cell adhesion molecules, and immunoreceptors, which induce proinflammatory and antimicrobial responses (Akira et al., 2006).

Despite the abundance of PG-containing microbiota and the numerous studies implicating PG as an immunostimulatory signal (Boneca, 2005), little is known about the systemic concentration of PG fragments in the environment or host, even though it is well documented that muropeptides serve also as signaling molecules and that a collection of receptor systems have evolved to detect these molecules.

### PG Sensors

In the last two decades, multiple structural motifs and proteins have been described to bind PG. Interestingly, not a single class of microorganism is sensed by only one type of receptor, hence ensuring a rapid and potent response while allowing for some specificity during, for example, infection. In this review, we summarize those receptors that recognize and bind PG.

### Lysin Motif

LysM (LysM) is considered a general PG-binding domain that binds specifically to molecules containing repetitions of NAG such as chitin, peptidoglycan, and short oligosaccharides (Buist et al., 2008; Mesnage et al., 2014). The LysM, usually 42–48 amino acids in length, is an ubiquitous modular cassette present across all kingdoms except for Archaea (Zhang et al., 2009). Usually, multiple motifs within one LysM domain are separated by spacing sequences (typically Ser-Thr-Asp/Pro) forming a flexible region in-between (Buist et al., 2008; Ohnuma et al., 2008). While it was initially identified in bacterial cell wall degrading enzymes [e.g., *E. coli* lytic tranglycosylase MltD (Bateman and Bycroft, 2000), *Enterococcus faecalis N*-acetylglucosaminidase AtlA (Mesnage et al., 2014), *B. subtilis* D,L-endopeptidase CwlS (Wong et al., 2014), or *Lactococcus lactic N*-acetylglucosaminidase AcmA (Steen et al., 2005)], LysM is also present in many other proteins involved in PG synthesis or remodeling (Buist et al., 2008; Buendia et al., 2018). The study of several proteins involved in bacterial peptidoglycan synthesis and remodeling has shown that even when PG peptide stems are not necessary for LysM binding, they modulate the binding affinity (Mesnage et al., 2014). In plants, the recognition of PG by LysM containing proteins initiates a signaling cascade that can suppress the host immune response (Gust, 2015). Furthermore, several LysM-containing proteins have been described to be involved in diverse processes, including recognition of bacteria, during bacteria-plant symbiosis, bacteriophage infection, and assembly of bacterial spores (Andre et al., 2008; Buist et al., 2008; Zipfel, 2014; Gust, 2015; Dworkin, 2018).

### PASTA Domain

**P**enicillin-binding **a**nd **S**er/**T**hr kinase-**a**ssociated (PASTA) proteins are essential tools for bacteria to sense and respond to the host environment and antibiotic stress as they play a central role in virulence and β-lactam resistance *via* their ability to regulate metabolism, cell division, and cell wall homeostasis through the recognition of muropeptides (Shah et al., 2008; Pensinger et al., 2018). The PASTA motif is involved in recognizing not only self-PG fragments but also exogenous muropeptides (Shah et al., 2008). Ligands are mostly species-specific, but a preference for muropeptides from species producing cell walls of similar composition (for example, containing *m*DAP in the third position in the peptide stem) has also been described (Lee et al., 2010; Mir et al., 2011).

### NOD-Like Receptors

Nucleotide binding and oligomerization domain proteins (NODs) are intracellular regulatory proteins that respond to a variety of signaling molecules including PG-derived fragments (Girardin et al., 2003c; Martinon and Tschopp, 2005; McDonald et al., 2005; Dziarski and Gupta, 2006; Strober et al., 2006; Le Bourhis et al., 2007; Sorbara and Philpott, 2011; Keestra-Gounder and Tsolis, 2017). NLRs show a conserved architecture, containing a C-terminal leucine-rich repeat domain, a central nucleotide binding and oligomerization domain, and N-terminal caspase activation and recruitment domain (Inohara and Nunez, 2003; Martinon and Tschopp, 2005). NOD1 and NOD2 are the best characterized NLRs, so far. NOD1 recognizes molecules containing D-Glu-*m*DAP (including PG free, mono-, and disaccharide peptides) (Girardin et al., 2003a), which are primarily found in Gram-negative bacteria with some exceptions such as *Bacillus* spp., Mycobacterium sp., *Listeria* spp., and *Lactobacillus plantarum* (Girardin et al., 2003c; Bourhis et al., 2007; Mahapatra et al., 2008; Bernard et al., 2011), while NOD2 senses NAM-D-Ala-D-Glu unit, ubiquitously present in both Gram-positive and Gram-negative mono- and disaccharide di-, tri-, and tetrapeptides (Girardin et al., 2003b; Dagil et al., 2016). PG fragments from non-invasive bacteria are transported into the eukaryotic cytosol through bacterial secretion systems, endocytosis, or specific membrane transport systems [PEPT: PepT1, PepT2, and pannexin (Vavricka et al., 2004; Charrier and Merlin, 2006; Kanneganti et al., 2007; Swaan et al., 2008)] or are delivered *via* OMVs (Philpott et al., 2014; Kaparakis-Liaskos and Ferrero, 2015; Canas et al., 2018), where they are sensed by both NOD receptors. The detection of PG by NOD proteins results in the activation of intracellular signaling cascades that triggers the nuclear factor-κB (NF-κB), innate response involved in inflammatory responses, and antimicrobial activity (Fritz et al., 2006; Meylan et al., 2006; Franchi et al., 2009).

### Peptidoglycan Recognition Proteins

Peptidoglycan recognition proteins (PGRPs) are evolutionarily conserved innate immunity molecules homologous to bacteriophage type 2 amidases found in animals and humans that present bactericidal activity (Dziarski, 2004; Royet and Dziarski, 2007). All PGRPs have a carboxy-terminal amidase domain (named PGRP domain) with a specific binding site for muramyl penta-, tetra-, or tri-peptides (Royet and Dziarski, 2007), but some mammalian PGRPs also have an additional binding site specific for bacterial lipopolysaccharide (Tydell et al., 2006; Sharma et al., 2011). So far, diverse PGRPs have been identified in insects [e.g., *Drosophila* has 13 PGRPs (Royet et al., 2011; Kurata, 2014)] and mammals [e.g., humans and mice have four (PGLYRP 1–4) (Liu et al., 2000; Lu et al., 2006; Cho et al., 2007; Diziarski and Gupta, 2010)] that recognize diverse PG fragments depending on their affinity and have a function in antibacterial immunity and inflammation. Instead of activating the innate system, PGRPs directly kill bacterial cells by binding PG, either to muramyl-peptides exposed by lytic endopeptidases in Gram-positive bacteria or uniformly to the outer membrane in Gram-negative bacteria (Kashyap et al., 2017). PGRP-PG interaction activates bacterial two-component systems (CssR-CssS and CpxA-CpxRin in Gram-positive and Gram-negative bacteria, respectively) that induce bacterial lysis by membrane depolarization and the simultaneous induction of oxidative, thiol, and metal stresses, which produce bacterial killing (Royet et al., 2011; Kashyap et al., 2014, 2017). Some data also suggest that the amidase domain acts as a scavenger to degrade PG and control the immune response (Mellroth et al., 2003).

### C-Type Lectin-Like Receptors

C-type lectin-like receptors (CTLRs) are a major class of PRR that present an extracellular carbohydrate recognition domain that putatively binds sugar moieties within the glycan backbone of bacterial PG or the fungal glucan mannan, in a calcium-dependent manner (Plato et al., 2013; Sukhithasri et al., 2013). Upon ligand recognition, specialized CTLRs trigger or inhibit a variety of signaling pathways, thus initiating pathogen phagocytosis, cytokine production, and activating diverse immune responses (Mayer et al., 2017). CTLRs bind to various pathogens, including viruses, fungi, parasites, and bacteria, and little is known about their specific role (if any) in PG detection. Regenerating gene family protein 3A (Reg3A) and mannose-binding lectin (MBL) protein are the only ones proven so far to bind PG (Sukhithasri et al., 2013). Reg3A is a lectin family protein that recognizes bacterial PG and presents bactericidal activity against Gram-positive bacteria (Lehotzky et al., 2010; van Ampting et al., 2012), while MBL is an oligomeric, calcium-dependent serum protein that recognizes both bacterial and fungal cell wall components leading to the activation of the lectin complement pathway (Shi et al., 2004; Nadesalingam et al., 2005).

### Hexokinases

Known as the first enzyme involved in glycolysis that catalyzes the phosphorylation of glucose to glucose-6-phosphate, hexokinases are also eukaryotic cytosolic sensors for PG. Recently, it has been suggested that the monomeric sugar NAG, generated during PG hydrolysis, can trigger the activation of the inflammatory programs in immune cells through binding and dissociating the cytosolic hexokinase (Gerriets et al., 2015; Wolf et al., 2016). Active hexokinases are associated with the mitochondrial outer membrane but are released when inhibited by NAG, similar to when glucose-6-phosphate (the product of hexokinase) accumulates the cytosol and promotes activation of the NLRP3 inflammasome, which regulates the processing and secretion of interleukin (IL)-1b and IL-18 (Shimada et al., 2010). Although the mechanism by which this occurs has not yet been described, a model has been proposed in which hexokinase acts as a pattern recognition receptor, alerting the cell to the degradation of bacterial PG in phagosomes and activating an inflammatory response *via* disruption of the glycolytic pathway and the mitochondrial function (Wolf et al., 2016).

## Mechanisms to Avoid PG Recognition

Bacteria have evolved sophisticated molecular strategies to subvert host defences by interfering with molecules involved in pathogen recognition and signaling. Both pathogenic and commensal bacteria are able to modify their PG in order to change the interaction with receptors and therefore avoid triggering the host immune responses (Boneca, 2005; Davis and Weiser, 2011). PG modifications fall into two main groups: (1) modification of glycan backbone to resist catalytic activity of PG-hydrolytic enzymes (*N*-deacetylation, *N*-glycolylation, O-acetylation) and (2) modifications of the stem peptides to evade immune recognition (L-Ala peptide substitutions, D-Glu and *m*DAP amidation, and *m*DAP substitution by L-ornithine). Thus, these modifications constitute a tactic that provides resistance to cell lysis and helps bacteria to evade the host immune system. For specificities about PG modifications, we refer to excellent reviews on the topic (Moynihan et al., 2014; Ragland and Criss, 2017; Yadav et al., 2018).

## Muropeptides as Signaling Molecules

**Figure 3:**
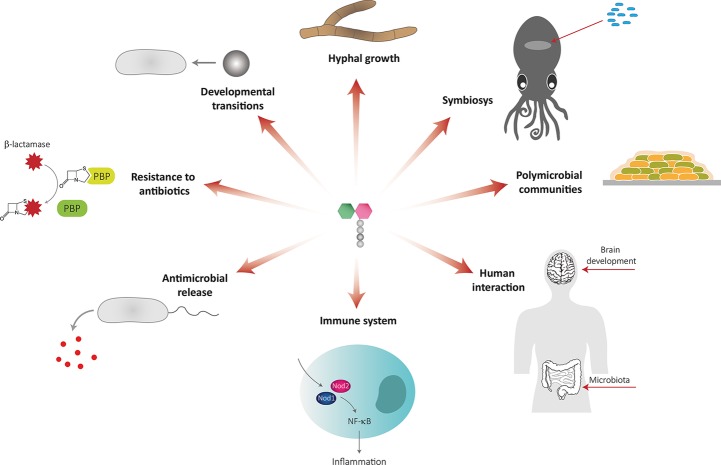
Muropeptides as signaling molecules.

PG remodeling produces soluble PG fragments that can have a role in bacteria-bacteria and bacteria-host communication and act as signaling molecules that trigger adaptive responses (Figure 3, Table 1).

**Table 1:** Messenger functions of muropeptides.

PG fragment	Structure	Sensing molecule	Function
Dissacharide tripeptide	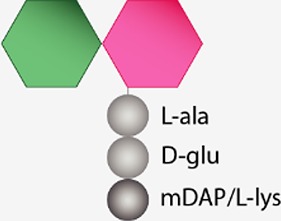	PrkC and homologs (STPKs)	Induction of germination (Shah et al., 2008; Dworkin and Shah, 2010) Exit from dormancy (Keep et al., 2006; Mukamolova et al., 2006)
*m*DAP-type PG (tri-, tetra-, pentapeptides)	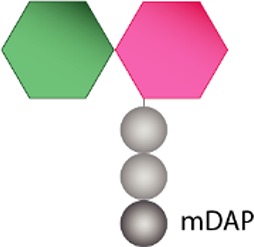	NA	Induction of rippling in *M. xanthus* (Shimkets and Kaiser, 1982)
Monomeric NAG sugar	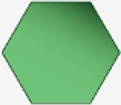	Ngt1 NA NA	Hyphal growth induction (Alvarez and Konopka, 2007) Antimicrobial induction in *P. aeruginosa* (Korgaonkar and Whiteley, 2011) and *Streptomyces coelicolor* (Rigali et al., 2006) CURLI fiber expression *in E. coli* (Konopka, 2012; Naseem et al., 2012)
Muramyl-dipeptide	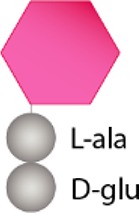	Cyr1p	Hyphal growth induction (Xu et al., 2008)
Anhydro-murotetrapeptide (Tracheal cytotoxin, TCT)	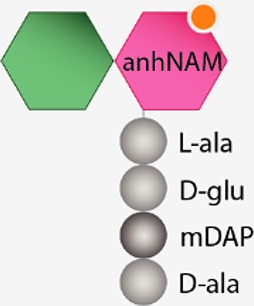	NA PGRP2 PGRP3-4	Signaling for morphogenesis (Koropatnick et al., 2004) Hydrolysis of pro-inflammatory PG fragments (Troll et al., 2010) Induction of inflammatory response (Goodson et al., 2005)
Anhydro-muramyltripeptide	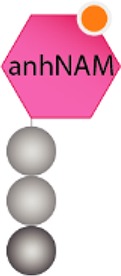	AmpR	β-Lactamase induction: AmpC (Uehara and Park, 2002, 2008)
Disaccharide pentapeptide	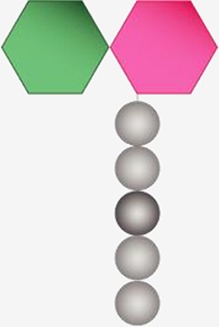	BlrB	β-Lactamase induction: Amp, Cep, Imi (Tayler et al., 2010)
Dipeptide	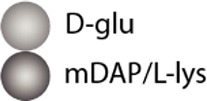	BlaI/MecI	β-Lactamase induction: BlaZ, BlaP, MecA (Amoroso et al., 2012)
Dipeptide D-Glu-*m*DAP (mono and disaccharide peptides containing this structure)	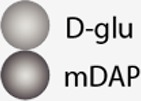	NOD1	NF-κB Innate response activation (Girardin et al., 2003a,b)
Muramyl-dipeptide (disaccharide di-, tri-, tetrapeptides)	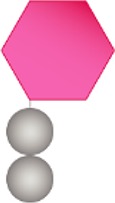	NOD2	NF-κB Innate response activation (Fritz et al., 2006; Meylan et al., 2006; Franchi et al., 2009)

### Developmental Transitions

Many bacteria have sophisticated mechanisms to undergo morphological changes in response to environmental stress including the formation of spores, dormant cells, persisters, or viable but non-culturable cells (Oliver, 2005; Wood et al., 2013; Li et al., 2014; Huang and Hull, 2017). Cells in these states monitor their environment seeking for improved conditions to reverse non-active states and reinitiate growth. Taking into account the quantity of PG release by bacteria (Doyle et al., 1988), it is plausible that muropeptides play a role as interspecies signal molecules to promote microbial growth under favorable conditions (Keep et al., 2006).

For example, *B. subtilis* spores are able to germinate in the presence of low concentration of muropeptides (disaccharide tripeptides containing *m*DAP) released by actively growing cells (Shah et al., 2008; Dworkin and Shah, 2010). Muropeptide-driven exit from dormancy requires the PrkC kinase, a member of the serine/threonine kinase (STPK) family, which has an intracellular kinase domain and an extracellular PG binding domain with multiple PASTA repeats (Yeats et al., 2002; Squeglia et al., 2011). PrkC binds PG, initiating a signal cascade that leads to spore germination (Shah et al., 2008). STPKs have a variety of roles in bacteria and are found in most Gram-positive bacteria. To date, PrkC homologues have been identified in *S. pneumoniae* (StkP), *S. mutans* (PknB), *M. tuberculosis* (PknB), *C. difficile* (PrkC), and *S. aureus* (PknB) (Fernandez et al., 2006; Sebaihia et al., 2006; Donat et al., 2009; Maestro et al., 2011; Mir et al., 2011). Interestingly, *S. aureus* PknB homolog responds not only to *m*DAP PG-type but also to the L-Lys-containing muropeptides (Dworkin and Shah, 2010), suggesting that bacteria expressing this kinase can react to signals from all species that produce PG.

Muropeptides are also implicated in the exit from dormancy of *Micrococcus* and *Mycobacterium* through the resuscitation-promoting factor (Rpf), a muralytic enzyme that cleaves the β-1,4 glycosidic bond in the glycan backbone of PG (Mukamolova et al., 2002, 2006). Combined with other hydrolytic enzymes, Rpfs might generate *m*DAP-containing muropeptides, which can bind to STPKs and trigger resuscitation in an analogous manner to spore germination in *B. subtilis* (Shah et al., 2008; Kana and Mizrahi, 2010). Furthermore, these *m*DAP-muropeptides might be detected by other receptors like Nod1 (Girardin et al., 2003a,c), suggesting that mycobacteria may, in addition, utilize those to modulate host innate immune responses during infection (Jo, 2008).

The predatory bacterium *Myxococcus xanthus* is able to respond to prey signals and alter its chemotactic and developmental pattern by forming fruiting bodies, where the vegetative cell differentiates into spores (Berleman et al., 2006; Keane and Berleman, 2016). One key phenomenon during fruiting body formation is the establishment of rhythmically advancing waves of cells (known as rippling) that has been shown to be induced by PG (Shimkets and Kaiser, 1982). This behavior is stimulated not only by *M. xanthus* PG fragments but also by a variety of proteobacteria and Gram-positive bacteria PG (e.g., *E. coli, B. subtilis*) (Shimkets and Kaiser, 1982).

Altogether, these findings suggest a pathway for bacterial resuscitation from a non-growing state *via* the detection of cell wall fragments in the environment, which seems to be a widely used strategy in the microbial world (Keep et al., 2006).

### Interspecies Interactions

PG fragments serve as signals in a range of host interactions (both pathogenic and symbiotic relationship of bacteria with plants and animals) and also in prokaryote-prokaryote encounters. For example, *Bacillus cereus* mediates commensalism with bacteria from the Cytophaga-Flavobacterium group in the soybean rhizosphere (Peterson et al., 2006). PG isolated from *B. cereus* stimulates the growth of *Flavobacterium johnsoniae in vitro*, pointing out to a beneficial relationship between these rhizosphere microorganisms. It has been suggested that *F. johnsoniae* secretes a cell wall degrading enzyme that permits the mobilization of *B. cereus* PG fragments as a carbon source for their growth, although the responsible enzyme and mechanism are still unknown (Peterson et al., 2006).

In *C. albicans*, muramyl dipeptides exhibit a potent hypha-inducing activity by directly binding to adenylyl cyclase Cyr1p LRR domain that stimulates cAMP production and subsequent hyphal growth (Xu et al., 2008). In addition, when *C. albicans* undergoes hyphal morphogenesis as a response to the presence of PG, *P. aeruginosa* is able to form a dense biofilm on the filamentous hyphal cells and kill them. Interestingly, *C. albicans* has developed a mechanism to protect itself by responding to the quorum factor 3-oxo-C12 homoserine lactone produced by *Pseudomonas*, which restricts its growth to a budding pattern that is not attacked by the bacteria (Hogan and Kolter, 2002; Naseem et al., 2012).

There are also several examples of PG fragments triggering the production of antimicrobial compounds within bacterial communities. *P. aeruginosa* is found in acute and chronic wounds forming a biofilm resistant to antimicrobials. Recent studies proved that exogenous NAG and other PG fragments derived from commensal Gram-positive bacteria elevate the virulence of *P. aeruginosa,* which is able to respond to the presence of PG fragments by producing pyocyanin, a potent antimicrobial phenazine (Korgaonkar and Whiteley, 2011; Korgaonkar et al., 2013). As *Pseudomonas* lives in polymicrobial communities, this mechanism might be of advantage to monitor surrounding microorganisms in order to eliminate competitors or provide additional nutrients for growth. In a similar way, NAG also induces the production of antimicrobials in the soil bacterium *Streptomyces coelicolor* (Rigali et al., 2006).

Another pathogenic bacterium, *E. coli*, also responds to NAG molecules derived from PG degradation by reducing CURLI fibers and type 1 fimbriae synthesis, both being essential for pathogenesis (Konopka, 2012; Naseem et al., 2012). In this case, regulation of these bacterial structures could balance the interaction between the pathogen and the host immune response, delaying the inflammation and allowing the dissemination of the bacteria within the host.

### Induction of Antibiotic Resistance

Some bacteria are able to induce β-lactamases expression in the presence of high levels of antibiotics (e.g., *Citrobacter freundii*, *P. aeruginosa*, and *Stenotrophomonas*), a phenomenon that is tightly linked to PG recycling (Dietz et al., 1997b; Zeng and Lin, 2013; Yin et al., 2014). In Gram-negative bacteria, two major mechanisms have been characterized: the AmpG-AmpR-AmpC pathway and the BlrAB two-component system. Regulation of the β-lactamase AmpC relies on the relative concentrations of cytoplasmic anhydromuropeptides. In the absence of β-lactam pressure, the UDP-muramyl-pentapeptide PG precursor is bound to the transcriptional regulator AmpR, inhibiting the expression of the *ampC* gene (Jacoby, 2009). However, in the presence of β-lactams, PG synthesis is blocked due to the inhibition of PBPs transpeptidase activity (Moya et al., 2009; Cho et al., 2014) leading to dysfunctionality of PBPs and resulting in an accumulation of anhydromuropeptides (mainly anhydro-muramyl-tripeptides) in the periplasm (Cho et al., 2014). This accumulation displaces UDP-muramyl-pentapeptide from AmpR (Uehara and Park, 2002, 2008), and thus, *ampC* is expressed and β-lactamases are secreted to the periplasm, where they hydrolyze the antibiotic (Holtje et al., 1994; Jacobs et al., 1994; Jacobs, 1997; Dietz et al., 1997a). Similarly, in *S. aureus* and *Bacillus licheniformis*, accumulation of cytoplasmic dipeptides, D-Glu-L-Lys or D-Glu-*m*DAP, respectively, is responsible for triggering the inactivation of BlaI/MecI repressors, leading to the synthesis of β-lactamases BlaZ, BlaP, and MecA (Amoroso et al., 2012). In *Aeromonas hydrophila,* β-lactamase production is regulated by a different system in which expression of AmpC is controlled through the two-component system BlrAB. Upon β-lactam exposure, disaccharide pentapeptides accumulate in the periplasm, inducing the autophosphorylation of BlrB, which phosphorylates BlrA, activating the transcription of the AmpC, Cep, and Imi β-lactamases simultaneously (Tayler et al., 2010). Additionally, the BlrAB two-component system (also known as CreBC) has been associated with β-lactam resistance in *P. aeruginosa* and *Stenotrophomonas maltophilia* (Moya et al., 2009; Huang et al., 2015).

In the past years, the use of combination therapy with β-lactams and vancomycin to treat methicillin-resistant *Staphylococcus aureus* (MRSA)-infected patients has caused the emergence of β-lactam-induced vancomycin-resistant MRSA (BIVR-MRSA). The presence of β-lactams inhibits PG biosynthesis, producing the accumulation of large amounts of PG precursors (specifically lipid II) with free D-Ala-D-Ala terminals that bind with vancomycin, depleting its concentration. Even if it is well characterized that the muropeptide NAG-NAM-L-Ala-D-Gln-L-Lys-(ɛ-amino-4Gly)-D-Ala-2Gly triggers vancomycin resistance (Ikeda et al., 2010), the mechanism underlying BIVR phenomenon remains to be elucidated.

Though induction mechanisms differ, all mentioned pathways are controlled by the amount of soluble PG fragments and so are linked to cell wall turnover and PG recycling.

## Bacteria-Host Interactions

The interaction of bacteria with host cells through PG signaling molecules is well known, and the PG-mediated responses have been characterized in the past years. In mammals, plants, and some insects, PG-derived fragments are recognized by the innate immune system and promote host defence against bacterial infections (Royet et al., 2011; Gust, 2015; Capo et al., 2016; Pashenkov et al., 2018; Wolf and Underhill, 2018), indicating that PG recognition is an evolutionarily conserved process.

PG is involved in establishing symbiosis during *Vibrio fischeri* colonization of the *Euprymna scolopes* squid light organ (Koropatnick et al., 2004). In this marine mutualism, the squid uses light produced by *V. fischeri* to avoid predators during its nocturnal behavior. Juvenile squids make use of the ciliated epithelial cells in the light organ to acquire the *V. fischeri* symbiont from the environment in each generation. Bacterial cells swim through ciliated ducts to gain access to deep crypt spaces, where they lose the flagellum and establish a permanent association with the host. Once colonized, *V. fischeri* releases tracheal cytotoxin (TCT, 1,6-anhydro-disaccharide tetrapeptides containing *m*DAP), which in synergy with LPS derivatives triggers the normal morphogenesis of the light organ. Morphogenesis involves the loss of ciliated epithelium, the shortening and eventual loss of appendages (Koropatnick et al., 2004, 2007; Brennan et al., 2014), and the reduction of mucus secretion (Nyholm et al., 2000), which prevents the entry of other bacteria. *E. scolopes* has four known PGRPs that are expressed in the light organ, which could be responsible for TCT sensing (Goodson et al., 2005; Royet et al., 2011). PGRP3 and PGRP4 are proposed to function as PG receptors on the surface of light organ cells, while PGRP2 is thought to be secreted into the lumen of the crypts helping maintain an appropriate level of *V. fischeri*. PGRP2 also hydrolyzes PG fragments preventing the inflammatory response activation, which allows the beneficial coexistence of the symbiont with the host (Troll et al., 2010).

The two Gram-negative pathogens *Neisseria gonorrhoeae* and *Bordetella pertussis* also release high amounts of muropeptides during infection: a mixture of 1,6-anhydrodisaccharide tri- and tetrapeptides (Sinha and Rosenthal, 1980, 1981) and TCT (Rosenthal et al., 1987), respectively. PG fragments are sensed by the intracellular NOD1 and NOD2 proteins that trigger the NF-κB and the mitogen-activated protein kinase pathways, finally stimulating the activation of the innate immune response, the release of proinflammatory cytokines, and cell damage. In *B. pertussis* infection, the release of TCT causes the death and detachment of ciliated cells from the epithelium of the trachea (Goldman et al., 1982; Heiss et al., 1993), while a similar pathology is observed during gonococcal infection of human fallopian tubes (Melly et al., 1984; Woodhams et al., 2013; Chan and Dillard, 2017). Likewise, detection of PG fragments of other pathogens (e.g., *Shigella* spp.) by NOD proteins can stimulate the innate immune response too (Philpott et al., 2000; Girardin et al., 2001; Nigro et al., 2008).

Moreover, it has been proposed that in humans, PG fragments might present other signaling functions apart from modulating the inflammatory response. Aside from defending the host against pathogens, the immune system is also involved in accommodating host colonization by symbiotic microorganisms and maintaining microbiota-host homeostasis. PG fragments are therefore part of the mechanism that controls interactions between the microbiota and host and has effects on host physiology and development outside the gastrointestinal system (Royet et al., 2011). Gut microbiota is a source of PG that can be translocated from the intestinal mucosa into circulation in the absence of pathogens (Clarke et al., 2010).

For example, recent studies strongly suggest that PG fragments from the intestinal microbiota have the potential to affect the immune system and govern the inflammatory response through NOD proteins (Hergott et al., 2016). In a similar way, and even though the underlying mechanisms remain to be elucidated, gut microbiota has been proposed to modulate brain development and behavior. PG fragments derived from commensal gut microbiota can be translocated into the brain by crossing the blood-brain barrier and can induce inflammation (Fillon et al., 2006; Arentsen et al., 2017). During mice brain development, PG fragments are sensed by pattern recognition receptors (PRRs) expressed during a specific temporal window, in concordance with the PG accumulation observed in the cerebellum and in parallel with the bacterial colonization process (Arentsen et al., 2017). Interestingly, any perturbation of the gut microbiota (e.g., antibiotic treatment) alters the expression of those PRRs in the brain, suggesting that this disruption may alter the developing brain, making it more susceptible to disorders or increasing the risk for immune diseases (Arentsen et al., 2017, 2018). Still, little is known about the structure of the PG molecule that generates these effects or the mechanism behind it.

Finally, somnogenic activity has been attributed to some PG fragments derived from gut microbiota, such as muramyl peptides containing DAP (Krueger, 1985; Krueger and Opp, 2016). According to the reported data, this somnogenic property is structure-dependent, with muramyl-tripeptide being the smallest active molecule able to induce sleep.

## Conclusions

The role of PG as a MAMP has long been recognized, especially as signaling molecules that modulate the innate immune response in some animals and plants. As during normal cell growth, bacteria release PG turnover products to the environment, and different organisms have developed sophisticated mechanisms to detect and respond to these molecules. Besides their role in infection or immune response development, bacteria use PG fragments as signaling cues to track the state of their cell wall or to monitor surrounding microorganisms. As many bacteria live within polymicrobial communities, this might be a beneficial mechanism to eliminate competitors or to obtain additional nutrients for growth. As a result of these functions, PG-fragments can be considered as signaling molecules.

The amount of PG turnover products released to the environment is dependent on the PG recycling pathway, and so, it is expected that bacteria regulate this process. Information regarding the regulation of AmpG or other transporters with similar activity is therefore crucial for a complete understanding of the function(s) of released anhydromuropeptides and other PG fragments; however, few studies have focused on this kind of protein (Cheng and Park, 2002; Chahboune et al., 2005; Zhang et al., 2010; Chan and Dillard, 2016; Li et al., 2016). A recently described assay to quantify AmpG-mediated transport (Perley-Robertson et al., 2016) may help to understand important aspects regarding the regulation of this permease in years to come. Likewise, other interesting tools have recently been developed to investigate PG recycling by studying other enzymes involved in this pathway (DeMeester et al., 2018).

The development of highly sensitive analytical methods and the use of synthetic muropeptides could help to elucidate the specific receptors that are able to bind PG as much as to determine the agonist PG structures and their role as signaling molecules. Overall, PG sensing seems to be a global mechanism that leads to different responses, so it is conceivable the existence of multiple PG-sensing pathways. More research is needed to clarify the different sensing mechanisms, to determine the interplay level of the different receptors (or to identify new ones), or understand the responses generated by muropeptides during bacteria-bacteria or bacteria-host interactions.

## Author Contributions

All authors listed have made a substantial, direct and intellectual contribution to the work, and approved it for publication.

### Conflict of Interest Statement

The authors declare that the research was conducted in the absence of any commercial or financial relationships that could be construed as a potential conflict of interest.
